# Involvement of the vertebral artery in hemifacial spasm: clinical features and surgical strategy

**DOI:** 10.1038/s41598-021-84347-x

**Published:** 2021-03-01

**Authors:** Seunghoon Lee, Junghoon Han, Sang-Ku Park, Jeong-A. Lee, Byung-Euk Joo, Kwan Park

**Affiliations:** 1 Department of Neurosurgery, Samsung Medical Center, Sungkyunkwan University School of Medicine, Seoul, Korea; 2 Neuroscience Center, Samsung Medical Center, Seoul, Korea; 3 Department of Neurosurgery, Konkuk University Medical Center, 120-1 Neungdong-ro, 17 Gwangjin-gu, Seoul, 05030 Korea; 4 Department of Neurology, Soonchunhyang University Seoul Hospital, Soonchunhyang University College of Medicine, Seoul, Korea

**Keywords:** Peripheral neuropathies, Movement disorders

## Abstract

The vertebral artery (VA)-involved hemifacial spasm (HFS) has distinctive clinical features and performing microvascular decompression (MVD) is challenging. We described the clinical presentations of VA-involved HFS and the outcomes of MVD using the interposition method. Between January 2008 and March 2015, MVD was performed in 271 patients with VA-involved HFS. Demographic characteristics, preoperative severity, intraoperative findings, spasm-free outcome, and complications were retrospectively evaluated. A control group of 1500 consecutive patients with non-VA-involved HFS was enrolled. VA-involved HFS was associated with older age (*p* < 0.001), less female predominance (*p* < 0.001), more left-sided predominance (*p* < 0.001), and rapid symptom progression before MVD (*p* < 0.001). The Teflon Fulcrum method allowed intraoperative identification of the neurovascular compression site in 92.6% of the cases, and showed more severe indentation on the facial nerve (*p* < 0.001). Changes in the brainstem auditory evoked potentials during MVD (*p* < 0.001) and postoperative non-serviceable hearing loss (*p* < 0.001) were more frequent in patients with VA-involved than in non-VA-involved HFS. The spasm-free outcome and overall complication rates after MVD were not significantly different between the groups. VA-involved HFS has distinctive clinical features and poses a major surgical challenge for MVD success. The interposition method is a feasible surgical strategy in VA-involved HFS.

## Introduction

Since Gardner^1^ and Janneta^2^ demonstrated and popularized microvascular decompression (MVD) in patients with hemifacial spasm (HFS), the advancements in microsurgical and neuromonitoring techniques have helped neurosurgeons to improve the cure rate of MVD to over 90%. Moreover, the rate of complications that can lead to permanent neurological sequelae has reduced to 1%^3–8^. Given that botulinum toxin provides only transient improvement of HFS symptoms and no durable, effective medical therapy has been found, successful MVD is of the utmost importance for the resolution of HFS^3^.

Visualization of the neurovascular compression (NVC) site at the root exit zone (REZ) of the facial nerve is essential during MVD for a successful outcome. Minimal cerebellar retraction to avoid neurovascular injury might not always allow complete exposure of the NVC site, which is a major cause of unsuccessful MVD^9^. The vertebral artery (VA) as an offending vessel for HFS exacerbates this potential difficulty. Due to its large caliber and augmented stiffness, the VA occupies most of the surgical field, is difficult to move, and obstructs the compression site near the REZ of the facial nerve. Previous studies have reported challenges in positioning prostheses between the nerve and the vessel (interposition method) and in performing various surgical techniques to completely mobilize the VA away from the facial nerve (transposition method). Recurrence of symptoms and failure of surgery due to inadequate decompression of the VA has also been reported^9–16^. However, several transposition methods to mobilize the VA are not entirely applicable to all cases and cannot be performed easily. Moreover, the number of case series with VA-involved HFS is limited and shows clinical results similar to the outcomes of non-VA-involved HFS, which are in contrast with previous studies^14,16–22^.

Therefore, this study aimed to elucidate whether clinical features, including spasm-free status and complications after MVD, differ between VA-involved and non-VA-involved HFS and whether performing MVD by the transposition method is necessary. We hypothesize that it is practical and rational to perform MVD by the interposition method in patients with VA-involved HFS if the clinical results are comparable to those achieved with MVD in patients with non-VA-involved HFS.

## Results

Of the 1771 HFS patients who underwent MVD between January 2008 and March 2015, the VA was involved as the offending vessel in 271 (15.3%) patients. The specific offending vessels in the VA and non-VA groups are shown in detail in Table 1. The VA with the anterior inferior cerebellar artery (58.7%) and the anterior inferior cerebellar artery alone (64.3%) were the most frequently involved offending vessels in each group, and most cases of VA-involved HFS (94.1%) involved multiple vessels with one vessel lying upon another vessel.

**Table 1:** Offending vessels in VA-involved and non-VA-involved HFS.

VA-involved HFS (N = 271)	N (%)	Non-VA-involved HFS (N = 1500)	N (%)
VA + AICA	159 (58.7)	AICA	965 (64.3)
VA + PICA	71 (26.2)	PICA	431 (28.7)
VA + AICA + PICA	25 (9.2)	AICA + PICA	99 (6.6)
VA only	16 (5.9)	Vein	5 (0.3)

*HFS* hemifacial spasm, *VA* vertebral artery, *AICA* anterior inferior cerebellar artery, *PICA* posterior inferior cerebellar artery.

### Clinical features of VA-involved HFS

The median age at surgery and the ratio of women to men among patients with VA-involved HFS were 55 years (48–60) and 150:121, respectively. The patients’ left sides were affected 6.3 times more often than their right sides. The median preoperative duration of symptoms was 36 months (21–60). Preoperative severity of the symptoms was evaluated according to a previously reported grading system^23^; 11.8% of the patients showed a severity of grade I; 43.9%, grade II; 32.8%, grade III; and 11.4%, grade IV. The patients were followed up for a median of 22.8 months (7.8–42.0) (Table 2).

**Table 2:** Demographics of patients with VA-involved HFS and non-VA-involved HFS.

	VA (N = 271)	Non-VA (N = 1500)	*p* value
Median age (years)	55 (48–60)	51 (43–58)	< 0.001
Sex (F:M)	150 : 121	1078 : 422	< 0.001
Affected side (right:left)	37 : 234	817 : 683	< 0.001
Median preoperative symptom duration (months)	36 (21–60)	42 (24–78)	< 0.001
**Preoperative severity of spasm**			0.377
Grade I	32 (11.8%)	129 (8.6%)	
Grade II	119 (43.9%)	678 (45.2%)	
Grade III	89 (32.8%)	499 (33.3%)	
Grade IV	31 (11.4%)	194 (12.9%)	
Median f/u duration (months)	22.8 (7.8–42.0)	22.0 (8.3–45.8)	0.901

*HFS* hemifacial spasm, *VA* vertebral artery, *M* male, *F* female, *f/u* follow-up.

During MVD, the NVC site was identified in 92.6% of the patients, and the degree of indentation in cases with the identified NVC site was mild in 8.4%, moderate in 41.0%, and severe in 50.6%. Intraoperative monitoring showed that abnormal muscle response (AMR) was disappeared after MVD in 96.6% of the patients, and the brainstem auditory evoked potential (BAEP) showed a change in 37.6% of the patients. The spasm-free rate at the last follow-up was 88.9%; 20.7% of the patients developed surgical complications. Immediate or delayed facial palsy was observed in 14.4% of the patients, and all of them recovered completely during the follow-up period. Hearing loss was observed in 3.7% of the patients, which was non-serviceable and permanent. Other complications included suspicious cerebrospinal fluid (CSF) leakage, wound infection, asymptomatic subdural hematoma, vocal cord palsy, and ageusia, which were observed in 4.8% of the patients. There was no definitive leakage of CSF or mortality (Table 3).

**Table 3:** Intraoperative findings and clinical outcomes of VA-involved and non-VA-involved HFS.

	VA (N = 271)	Non-VA (N = 1500)	*p* value
**Intraoperative findings**			
Identification of NVC site	251 (92.6%)	1349 (89.9%)	0.205
Degree of indentation			< 0.001
Mild	21 (8.4%)	247 (18.3%)	
Moderate	103 (41.0%)	601 (44.6%)	
Severe	127 (50.6%)	501 (37.1%)	
AMR positive	235 (86.7%)	1297 (86.5%)	
Post-MVD disappeared	227 (96.6%)	1263 (97.4%)	0.646
BAEP change	102 (37.6%)	392 (26.1%)	< 0.001
**Clinical outcomes after MVD**			
Spasm-free	241 (88.9%)	1330 (88.7%)	0.900
Complication^a^	56 (20.7%)	275 (18.3%)	0.412
Facial palsy^b^	39 (14.4%)	211 (14.1%)	0.963
Non-serviceable hearing loss^c^	10 (3.7%)	13 (0.9%)	0.001
Others^d^	13 (4.8%)	61 (4.1%)	0.580

*HFS* hemifacial spasm, *VA* vertebral artery, *NVC* neurovascular compression, *AMR* abnormal muscle response, *BAEP* brainstem auditory evoked potential, *MVD* microvascular decompression.

^a^A patient might have more than one complication.

^b^Facial palsy included both immediate and delayed type.

^c^The hearing loss included a sensorineural type only.

^d^Others included wound infection, aseptic meningitis, suspicious or definite cerebrospinal fluid leakage, vocal cord palsy, asymptomatic subdural hematoma, transient abducens nerve palsy, and ageusia.

### Comparison between VA-involved HFS and non-VA-involved HFS

A total of 1500 patients with non-VA-involved HFS were evaluated for the same parameters as those for the VA-involved HFS patients. The results of the comparison between the VA and non-VA groups are shown in Tables 2 and 3. Older age (*p* < 0.001), less female predominance (*p* < 0.001), and more left-side predominance (*p* < 0.001), and shorter duration of symptoms before MVD (*p* < 0.001) were seen in the VA group than in the non-VA group, and these differences were statistically significant. The distribution of spasm severity were not significantly different between the two groups. During surgery, the rate NVC site exposure was similar in the two groups (*p* = 0.205), and more severe indentation (*p* < 0.001) was found in the VA group. While the rate of the disappearance of AMR did not differ significantly between the two groups (*p* = 0.646), the BAEP change (*p* < 0.001) was more frequent in the VA group. The spasm-free rates at the last follow-up (*p* = 0.900) and the overall complication rate (*p* = 0.412) were not significantly different between the VA and non-VA groups. Postoperative non-serviceable hearing loss was permanent and more frequent in VA-involved HFS (*p* = 0.001).

## Discussion

The VA is characterized by a large caliber and higher stiffness, which makes it challenging for the neurosurgeon to mobilize it and decompress the facial nerve. Hence, the outcomes of MVD surgery in patients with VA-involved HFS are poorer than those in non-VA-involved HFS^17,18^. If the VA exhibits a dolichoectatic change, the procedure and outcomes become even more complicated. With the largest sample of patients with VA-involved HFS, we elucidated that VA-involved HFS was associated with older age and less female predominance and left-sided predominance. The preoperative symptom duration in VA-involved HFS was shorter, which indicates rapid symptom progression before MVD surgery in this group than in the non-VA group. Using the interposition Teflon Fulcrum method, clinical outcomes determined by spasm-free status and complications were similar among patients with VA-involved and non-VA-involved HFS.

The characteristics of VA-involved HFS in the present study are in line with the findings of previous investigations; 94.1% of the cases had multiple vessel involvement, including the VA, and all contributing vessels were indicated for decompression to ensure a spasm-free outcome. While most prior studies showed the influence of age and less female predominance, some of them did not find a statistically significant difference, probably due to small sample sizes. The higher incidence of cerebrovascular disease among men and in old age might explain these phenomena. Left-sided predominance is a considerably consistent feature across the literature, which might be due to several reasons: a variant of the left VA originating directly in the aortic arch, a higher percentage of left-dominant VA, and higher flow velocity and volume in the left VA^24,25^. We also hypothesize that strong pulsatile pressure from the VA positively influences preoperative severity of spasm; however, no statistical significance was found. Nevertheless, we observed that indentation on the facial nerve in VA-involved HFS was more significant than that in non-VA-involved HFS.

Despite the hypothetical assumption of a poor outcome in patients with VA-involved HFS and previous evidence supporting this hypothesis^18^, similar clinical outcomes achieved in patients with VA and non-VA-involved HFS have been recently reported^16,19–21^. With our large sample, we observed similar spasm-free rates between the two patient populations during the median of 22.8 months of follow-up. However, while statistically insignificant, overall complications after treatment occurred more frequently in the VA-involved HFS group. Moreover, higher rates of BAEP changes and permanent non-serviceable hearing loss were seen in patients with VA-involved HFS. Thus, MVD in patients with VA-involved HFS might involve a higher potential surgical risk.

Transposition of the vessel in MVD surgery might be theoretically ideal, and the outcomes from a case series using the transposition method showed a 100% spasm-free rate. However, there is no strong evidence about the superiority in clinical outcome achieved with the transposition method; this might be due to the small numbers of cases analyzed in previous studies^14,22^. Several transposition methods to mobilize the VA have been introduced: a Prolene sling technique^11^, VA anchoring with aneurysmal clip^10,13^, double-stick tape method with TachoComb^12^, stitched sling retraction technique^15^, bioglue-coated Teflon sling technique^14^, and a synthetic vascular graft sling technique^26^. However, they are surgically more challenging than the interposition method and are not applicable for all cases; in fact, they might be dangerous if many perforators are involved. It is often difficult to mobilize the VA away from the facial nerve and maintain the detached space between the nerve and the vessel using suction or the microsurgical elevator after mobilization. Moreover, complications related to iatrogenic vascular injury or thromboembolic cerebral infarction can occur due to mobilization of the VA with excessive force.

We explored the interposition method and developed the relatively simple microsurgical technique, Teflon Fulcrum method, to ease the mobilization of the VA, widen the surgical space for better visualization of the NVC site, and decompress the VA from the facial nerve. This technique can be used with the transposition method as well. During the process of transposing the VA away from the facial nerve, the Teflon fulcrum can facilitate mobilization of the VA and visualization of the NVC site and maintain the VA position when it is lifted over the facial nerve close to the petrous bone.

The clinical outcome of VA-involved HFS can be as good as that of non-VA-involved HFS when the MVD is performed properly using the interposition Teflon Fulcrum method. Although direct comparison between the interposition and transposition methods in VA-involved HFS might provide stronger evidence for the optimal surgical method, the present study provides evidence for the adequacy and success of the interposition method. We consider that it is feasible and reasonable to perform a safer interposition method in VA-involved HFS if clinical results of MVD similar to those of non-VA-involved HFS, to which the majority belong, can be achieved with the interposition method. However, this singular consideration of the interposition method may also mark the limitation of our study; we neither disregard the theoretical capacity of the transposition method when applied to MVD to achieve a 100% cure rate^27^, nor the 10% chance of MVD failure by the interposition method.

## Conclusions

VA-involved HFS has distinctive clinical features that are absent in non-VA-involved HFS. VA-involved HFS is a major surgical challenge for neurosurgeons performing MVD. Surgical outcomes achieved with the interposition Teflon Fulcrum method are similar between patients with VA-involved and non-VA-involved HFS. The interposition method can be recommended as a feasible surgical strategy when performing MVD to resolve VA-involved HFS.

## Methods

Of all the patients with HFS who underwent MVD between January 2008 and March 2015, the VA was involved as the offending vessel in 271 patients. During the same period, 1500 patients with non-VA-involved HFS were recruited consecutively as a control group. All surgeries were conducted by a single neurosurgeon in a single institution. The medical charts of patients were reviewed for preoperative severity grade of HFS^23^, symptom duration, operative technique, intraoperative finding, and postoperative outcome. We assessed facial palsy and hearing loss according to the House-Brackmann facial grading system^28^ and Gardner–Robertson hearing classification scale^29^, respectively. Facial palsy referred to facial dysfunction of grade II or higher, and non-serviceable hearing loss referred to pure tone audiometry (PTA) of 51 dB or higher or speech discrimination score of 50% or lower. PTA and speech audiometry (SA) were performed on all candidates for MVD and were repeated within 3–7 days after surgery. The hearing loss included a sensorineural type only. This study was approved by the institutional review board of Samsung Medical Center (no. 2017–11-028), which waived the requirement for informed consent because this is a retrospective study and the validity of the study would not be affected by the absence of patient consent. Moreover, no additional risk to the patient’s safety was expected by conducting this study without patient consent. All methods followed the relevant guidelines and regulations of the mentioned committee.

### Operative procedure and intraoperative findings

All MVD surgeries were performed using the interposition method, and the surgical procedure is described in previous literature^30^. The intraoperative BAEP, facial electromyography, and AMR were closely monitored during each surgery^31,32^. We classified the degree of indentation by the offending vessel on the facial nerve into three grades; grade 1 or mild indicating only traces of vascular compression, grade 2 or moderate, an evident indentation on the facial nerve, and grade 3 or severe, prominent indentation with discolorationy^33^.

### Teflon fulcrum method

**Figure 1:**
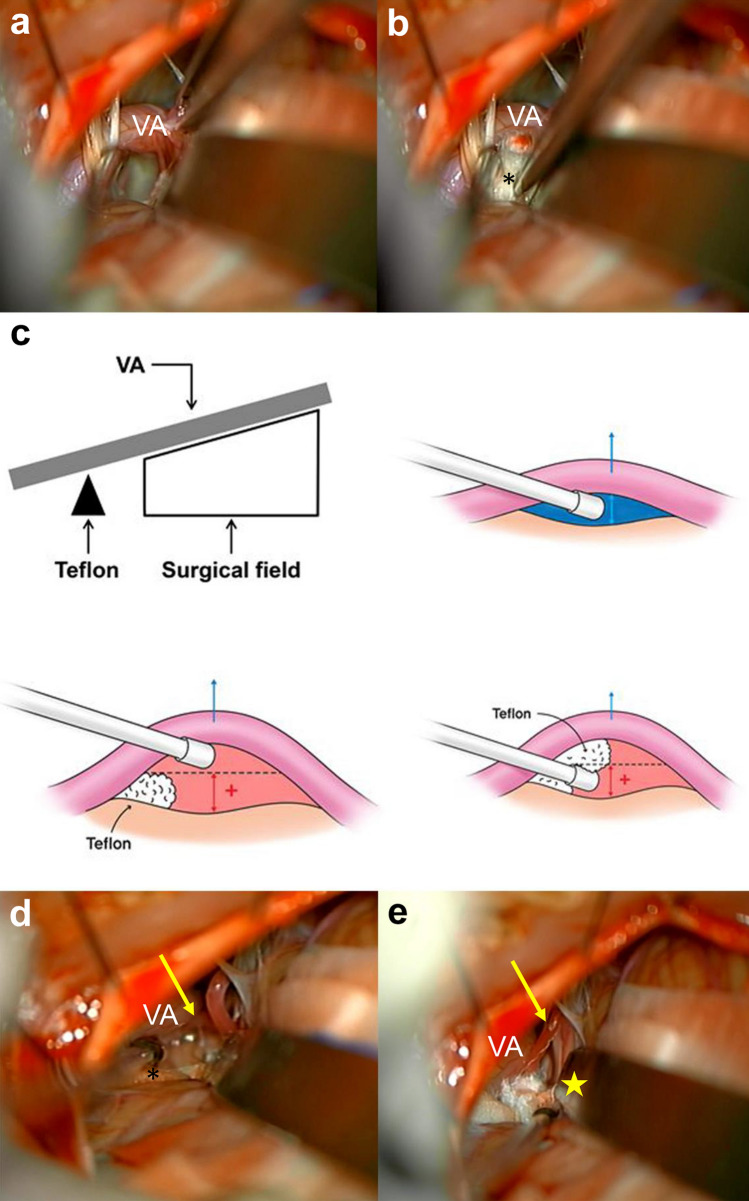
Step-by-step intraoperative photographs during microvascular decompression (MVD) for vertebral artery (VA)-involved hemifacial spasm with an illustration of the Teflon Fulcrum method. (**a**) Attempts to lift the VA high enough toward the petrous bone to expose the root exit zone using a suction tip failed. (**b**) Teflon felt (*) is placed between the proximal VA and ventromedial brainstem near the lower cranial nerves. (**c**) The inserted piece of Teflon works as a fulcrum, and the VA could be elevated away from the brainstem and maintained at the height of Teflon’s thickness. This allowed larger surgical space by either moving the VA distal to the fulcrum or pushing the fulcrum together with the overlying VA. (**d**) The mobilized VA with the support of the Teflon fulcrum from underneath provides a wider view of the surgical field. (**e**) The co-offending vessel (arrow), the anterior inferior cerebellar artery, underneath the VA and the neurovascular compression site (star) could be visualized by slightly adjusting the angle of microscope. Complete MVD was achieved by inserting one or two more Teflon felts. *MVD* microvascular decompression, *VA* vertebral artery.

After opening the dura, gentle cerebellar retraction allowed arachnoid dissection around the lower cranial nerves. Further cerebellar retraction facilitated the visualization of the REZ of the facial nerve at the ventromedial portion of the pontomedullary junction. However, the VA was identified in the microscopic surgical field by pushing the lower cranial nerves toward the neurosurgeon and obstructing the REZ in VA-involved HFS. Attempts to lift the VA toward the petrous bone, high enough to expose the REZ using a microsurgical elevator or suction tip, often failed (Fig. 1a). Since the VA itself is usually a large structure occupying most of the surgical field and is stiffer than the smaller vessels, such as the anterior inferior cerebellar artery or posterior inferior cerebellar artery, it could not be mobilized easily.

The Teflon Fulcrum method begins with the placement of a large piece of Teflon felt between the proximal VA and the ventromedial brainstem near the lower cranial nerves (Fig. 1b). The inserted piece of Teflon functions as a fulcrum, and the VA can be elevated away from the brainstem and maintained at the height of the Teflon’s thickness. A surgical space can then be secured by either moving the VA distal to the fulcrum or pushing the fulcrum together with the overlying VA to elevate the VA further (Fig. 1c). The mobilized VA, with the support of the Teflon fulcrum from beneath, provides a wider view of the surgical field with less effort than VA mobilization executed with the microsurgical elevator alone (Fig. 1d). The co-offending vessel underneath the VA and the NVC site can eventually be observed by slightly changing the angle of the microscope. Complete MVD was achieved by inserting one or two more Teflon pieces without inducing further neurovascular damages (Fig. 1e). Although we technically used the interposition method, we placed the Teflon felt between the VA and the facial nerve proximal or distal to the NVC site; hence, the site was free of the offending vessel and Teflon felt.

### Statistical analysis

Continuous variables were presented as median (interquartile range), and categorical variables were presented as number (percentage). To compare the demographics, intraoperative findings, and clinical outcomes of VA-involved and non-VA-involved HFS, the chi-square test and the Mann Whitney test were used. The result was considered statistically significant if the *p* value was < 0.05. All statistical analyses were performed using Statistical Analysis System (SAS^®^) software, Version 9.4 of the SAS System (SAS Institute, Cary, NC, USA; http://www.sas.com/) by the institutional biostatistics team.

## Abbreviations

MVD: Microvascular decompression
HFS: Hemifacial spasm
BAEP: Brainstem auditory evoked potential
NVC: Neurovascular compression
REZ: Root exit zone
VA: Vertebral artery
CSF: Cerebrospinal fluid
AMR: Abnormal muscle response
